# Atopic dermatitis: real-life experience with tralokinumab after dupilumab failure: a case series^☆^

**DOI:** 10.1016/j.abd.2023.09.009

**Published:** 2024-06-03

**Authors:** Patrícia Amoedo, Gilberto Rosa, Teresa Baudrier, Ana Filipa Pedrosa, Maria João Cruz

**Affiliations:** a Department of Dermatology and Venereology, Centro Hospitalar Universitário de São João, Porto, Portugal; b Faculty of Medicine, Universidade do Porto, Porto, Portugal; c Centro de Investigação em Tecnologias e Serviços de Saúde, CINTESIS, Porto, Portugal

*Dear Editor,*

Atopic dermatitis (AD) is a common chronic inflammatory skin disease, requiring safe and effective long-term treatments. Classic treatments had limited efficacy and significant adverse effects, but the growing knowledge of pathophysiology enabled the development of new target therapies that changed the paradigm.^1^ Two major classes emerged, biologics, which include monoclonal antibodies anti-IL-4Ra (dupilumab) and anti-IL-13 (tralokinumab), and JAK inhibitors (iJAK).^2^ Both groups had shown sustained efficacy and safety in moderate-to-severe AD, but there are no head-to-head studies, and data on patients who have received prior targeted therapies are limited.^2^, ^3^, ^4^

Here we report three cases of severe AD, with a mean baseline EASI of 35.9 (25.8–42.9), aged between 24‒32 years, refractory to multiple classical therapies, dupilumab and, in one case, also baricitinib, successfully treated with tralokinumab. Cases 1 and 3, failed to achieve EASI-75 after 6 months under dupilumab (300 mg, biweekly). A switch to baricitinib, the only approved alternative at the time, was made in Case 3, without a satisfactory response. In Case 1, cardiovascular risk factors and latent tuberculosis, contraindicated baricitinib, so a weekly dupilumab dosage was tried, without success. In Case 2, a rapid response was achieved after only 3 months (EASI-85), but there was a secondary failure at 9 months. Then, a switch to tralokinumab (300 mg, biweekly) was made in all patients, under a national early access program. A global improvement in symptoms and quality of life was observed in all patients and an EASI-90 was achieved within 6 months. Case 2 had an early response, reaching EASI-95 at 12-weeks (Figure 1, Figure 2, Figure 3). All patients are still under treatment, with a mean follow-up of 57 weeks. No adverse effects, including conjunctivitis, were reported.

**Figure 1:**
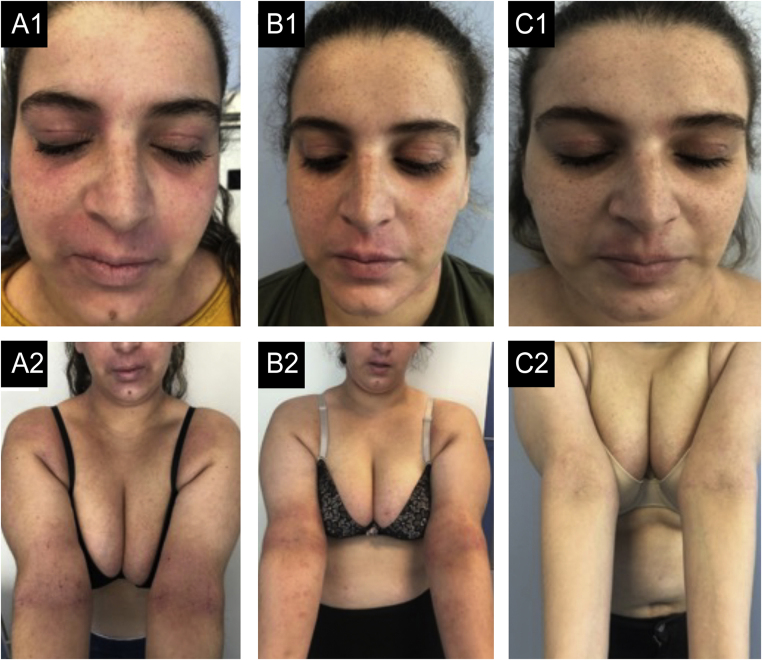
Case 1. (A1, A2) basal state: Severe eczema, affecting mainly the face, neck, and skin folds. There is severe erythema, papulation with some oozing and crusting and lichenification in the upper lip and skin folds. (B1, B2) After 12-months of dupilumab: Slight improvements, even after 6-months of weekly treatments. (C1, C2) After 6-months of tralokinumab: Almost clear skin, remaining only a small plaque in the upper lip, with mild erythema and lichenification and some residual hyperpigmentation.

**Figure 2:**
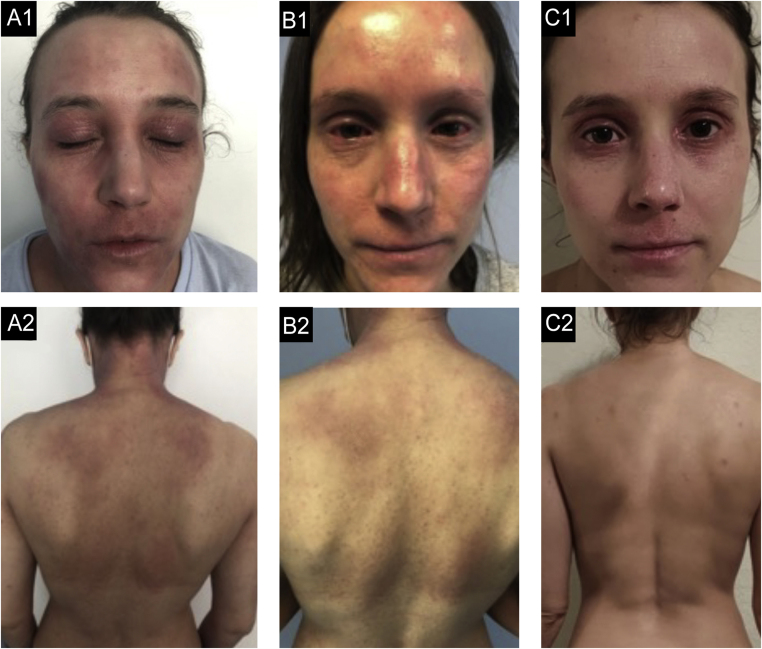
Case 2. (A2, A2) basal state: Severe eczema, widespread, with severe erythema, moderate papulation with oozing and crusting and marked palpebral lichenification. (B1, B2) After 9-months of dupilumab: Secondary failure after an initial improvement with partial response at 6-months with development of severe bilateral conjunctivitis. (C1, C2) After 3-months of tralokinumab: Global improvement, with almost clean skin, with residual lichenification in both eyelids and upper lip.

**Figure 3:**
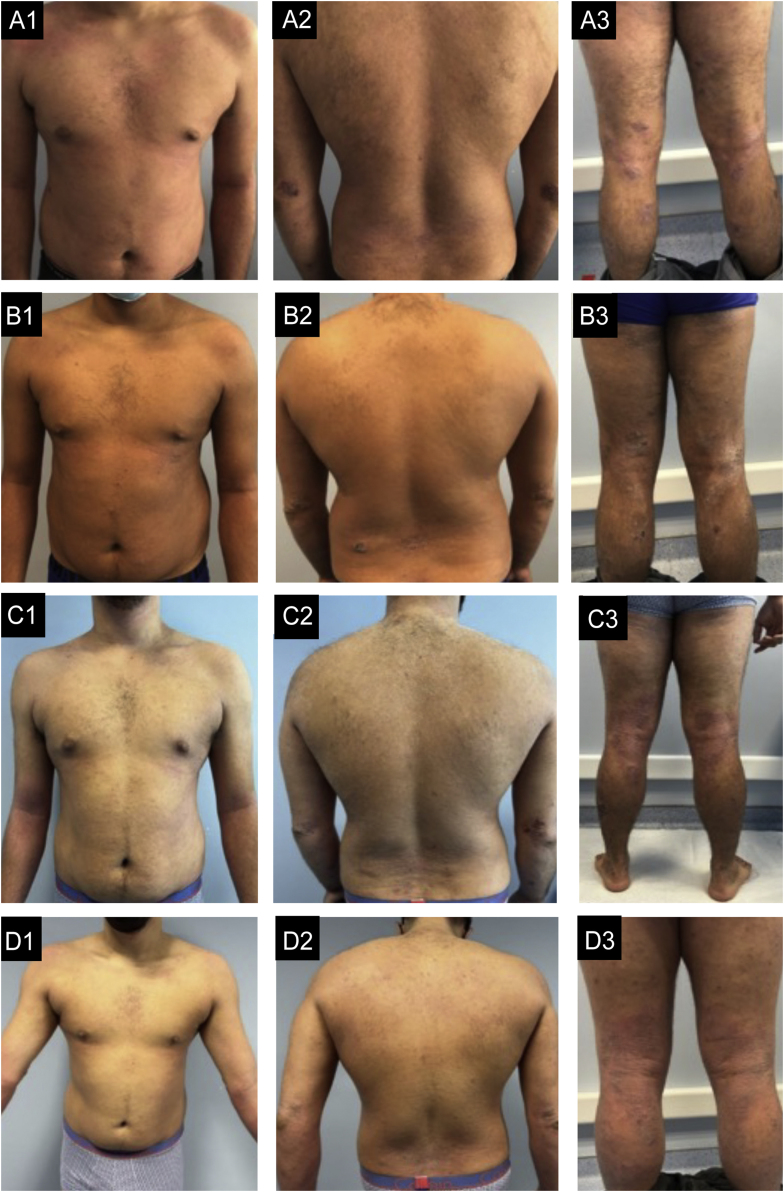
Case 3. (A1‒A3) basal state: Severe eczema, widespread, with areas of extensive moderate erythema and edema in the trunk and areas of nummular configuration with intense oozing and crusting and excoriation in the elbows, buttocks, thighs, and legs. (B1‒B3) After 6-months of dupilumab: Very small improvement. (C1‒C3) After 6-months of Baricitinib: No improvement with new lesions in the feet. (D1‒D3) After 4-months of tralokinumab: Global improvement, more evident in the trunk, with almost clear skin. In the limbs, edema and lichenification are less severe and there are no excoriations, which reflects an important improvement in pruritus.

AD is a Th2-mediated disease, involving both IL-4 and IL-13.^1^, ^3^, ^5^, ^6^ IL-4 was considered the pivotal cytokine, but recent studies showed overexpression of IL-13 in lesional skin, particularly in the chronic stage, with a correlation between its tissue levels and disease severity, suggesting a preponderant role in the underlying inflammation.^1^, ^5^, ^6^ In contrast, IL-4 levels were almost undetectable. Additionally, IL-13 has important implications in skin biology, skin microbiome, epidermal barrier, and inflammatory cell recruitment.^5^

Both dupilumab and tralokinumab, have been shown to be safe and effective in clinical trials, but there are no head-to-head comparison studies.^1^, ^6^ Systematic reviews with meta-analysis point to the superiority of iJAK, followed by dupilumab and tralokinumab. However, most trials are performed in monotherapy, with a maximum follow-up time of 20 weeks, which does not reflect daily practice.^7^ Additionally, refractory cases like ours, are usually excluded.^8^ Regardless, meta-analysis conclusions should be taken cautiously, as disparities between trial methodologies limit their comparability.^1^, ^3^, ^6^, ^7^ In fact, a recent open-label extension trial showed that in the long-term, tralokinumab results match those of dupilumab and iJAK, which is significant, considering AD chronicity.^9^ Two real-life case series with tralokinumab reported results consistent with those of the clinical trials, but patients who received prior target therapies, presented significantly worse responses.^8^, ^10^

In our cases, there was an excellent response to tralokinumab, despite the previous failure of dupilumab and, in one case, also baricitinib. In part, this may be due to a longer follow-up time, as most patients in the real-life series had less than 20 weeks of follow-up.^10^

Thus, despite the additional IL-4 inhibition by dupilumab, there seems to be a subset of patients that respond better to tralokinumab. Pharmacokinetic and pharmacodynamic differences may partly explain this, but there are patient factors that we need to unravel in order to make individualized therapeutic decisions.^8^

In conclusion, tralokinumab can be effective in patients unresponsive to other systemic treatments including dupilumab, with a good tolerability profile, but comparative studies are still necessary to guide clinician decision-making.

## Financial support

None declared.

## Authors’ contributions

Patrícia Amoedo: Writing and editing (lead).

Gilberto Rosa: Review (support).

Teresa Baudrier: Review (support).

Ana Filipa Pedrosa: Review and final approval.

Maria João Cruz: Review and final approval.

## Conflicts of interest

None declared.
